# Correlates of Sexual Function in a Sample of Spanish Women with Endometriosis

**DOI:** 10.3390/jcm10214957

**Published:** 2021-10-26

**Authors:** Ernesto González-Mesa, Davinia Moya-Bejarano, Carmen Aisha Butrón-Hinojo, Pilar Marín-Sánchez, Marta Blasco-Alonso, Jesús Salvador Jimenez-López, Emilia Villegas-Muñoz, Daniel María Lubián-López

**Affiliations:** 1 Department of Obstetrics and Gynecology, School of Medicine, University of Malaga, 29071 Málaga, Spain; 2 Department of Obstetrics and Gynecology, Regional University Hospital of Málaga, 29011 Málaga, Spain; martablascoalonso@gmail.com (M.B.-A.); jesuss.jimenez.sspa@juntadeandalucia.es (J.S.J.-L.); evillegasm@sego.es (E.V.-M.); 3 Department of Obstetrics and Gynecology, Punta de Europa Hospital of Algeciras, 11207 Cádiz, Spain; franciscoj.martos.sspa@juntadeandalucia.es; 4 Department of Obstetrics and Gynecology, University Hospital Virgen de la Arrixaca, 30120 Murcia, Spain; udmog_sms@carm.es; 5 Department of Obstetrics and Gynecology, University Hospital of Jerez de la Frontera, 11407 Cádiz, Spain; dmlulo@gmail.com

**Keywords:** endometriosis, female sexual function, dyspareunia, psychosocial factors

## Abstract

Background: In the present study, we aim to report on the sexual function of women experiencing symptoms of endometriosis, analysing the clinical and psychosocial factors that may be associated. Methods: A multicentre cross-sectional study was performed to analyse the sexual function in a sample of 196 Spanish women with endometriosis, using the Female Sexual Function Inventory. Results: The Female Sexual Function Inventory (FSFI) was validated in our endometriosis study group. The mean FSFI score for the sample was 22.5 (SD 6.6), with 20.9 and 26.9 being in the 25th and 75th percentiles, respectively. Although physical sexual pain and dyspareunia were factors that influenced the sexual function of women with endometriosis, our results show that the impairment was multifactorial. Conclusions: We found impaired sexual function in women diagnosed with endometriosis. The final model included deep endometriosis, depression, age, and unemployment as strongest predictive factors for poor (deteriorated) sexual function.

## 1. Introduction

Endometriosis is known to affect 10% of premenopausal women and 30–50% of those with chronic pain or infertility [1,2,3]. Endometriosis is an enigmatic disease of yet-unknown origin and pathogenesis. It is sustained by theories from long ago, when Sampson [4] described it as ectopic implants of menstrual shredding passed to the abdominal cavity through the Fallopian tubes. Recently, Brosens and Benagiano [5] suggested that it starts with neonatal hormonal deprivation bleeding that many newborn girls express in a retrograde fashion. Implants would remain until puberty. A celomic theory states that embryonic cells from the Müllerian ducts persist in ectopic locations. At puberty, stimulated by oestrogens, they grow to build up endometriotic lesions [6]. For other authors, endometriosis is a “heritable, hormone-dependent gynecological disorder”; Nyholt’s metanalysis [7] identified five novel loci related to the risk of developing endometriosis. All five are involved in sex steroid pathways.

Endometriosis is a benign chronic inflammatory and oestrogen dependent disease, defined by the presence of endometrial gland and stroma-like tissue outside of the uterus (Ballard et al., 2008; Berek et al., 2012). It is one of the most common gynaecological diseases. Endometriosis is known to affect 10% of premenopausal women and 30–50% of those with chronic pain or infertility [1,2,3].

The diagnosis is based on the woman’s history, symptoms, and signs. It is corroborated by physical examination and imaging techniques and finally proven by histology [2].

Laparoscopy is the “gold standard” for the diagnosis of endometriosis. Surgical biopsies allow histological confirmation. Laparoscopy should be performed preferably by experienced surgeons. Removal of all disease present must be accomplished in the same procedure. 

Medical management is a centrepiece. According to the consensus, old-time favourites such as danazol or gestrinone should be used only in the absence of side effects when other treatments have proven ineffective [8]. Progestogens have proven efficacy [9], whereas gonadotropin-releasing hormone (GnRh) agonist therapy is not recommended for long-term use. Oral progestin-only pills have demonstrated their ability to control the extent of endometriotic lesions on a long-term basis. Combined oral contraceptives (OCs) provide initial pain relief, but the long-term efficacy as a treatment for endometriosis lacks clinical evidence [10]. Moreover, there are even some data supporting potential adverse effects on the progression of the disease. Newly introduced oral GnRh antagonist elagolix NR is associated with few minor side effects (hot flashes), excellent reduction of endometriosis-associated pain, and arrest of the progression of the disease when used for an extended period of 12 months [11,12]. Surgery should be considered, during laparoscopy, in the treatment of the disease. All lesions presents should preferably be resected. The issue of endometriomas, a never-ending dilemma, is discussed with sound evidence from the recent literature. 

Infertility treatments for patients with endometriosis need special consideration. Surgery and assisted reproduction techniques (ARTs) cross over according to the different stages of the disease and the patient’s age. Minimal and mild disease frequently benefit from expert surgery. Advanced moderate and severe stages usually require in vitro fertilization (IVF). DIE should be treated only by expert surgeons, preferably by interdisciplinary teams. The question of whether it should be operated before infertility treatments remains controversial [13].

There is no confirmed correlation between extended disease and severity of symptoms, reproductive prognosis, or recurrence of pain [14] and the classification systems in current use continue having poor correlation with disease symptoms [15]. Most of the lesions of endometriosis are located in the posterior pelvic cavity [16]. These lesions form hard nodules in the uterosacral ligament, uterine rectal depression, and vaginal fornix. During sexual intercourse, these nodules are affected by an external impact force.

The quality of sexual life plays an important role in the overall quality of life. Approximately 70 million adult and adolescent women worldwide suffer from endometriosis. More than 70% of patients with endometriosis have obvious pain symptoms, such as dyschezia, chronic pelvic pain, sexual intercourse pain, and faecal pain. Approximately two thirds of women with endometriosis have sexual dysfunction that is not limited to deep dyspareunia [17]. Quality of life and mental health of women are significantly negatively affected by dyspareunia [18,19]. Dyspareunia is a complaint of 32–70% of women with endometriosis (De Graff et al., 2013). Nevertheless, the presence of pain at penetration is not the only determinant of these women’s sexual health (Melis et al., 2015). Studies on the sexual function of women with chronic diseases showed that these affect multiple domains of sexual function (Rosen et al., 2006). An increasing amount of attention has been paid to female sexual dysfunction by women and clinicians in recent years. In 1998, the American Urinary Foundation defined female sexual dysfunction as women who are unable to participate in the desired sexual behaviour. In female sexual dysfunction, there is difficulty in satisfaction or even a lack of satisfaction in the process of sexual behaviour. These feelings include loss of libido, arousal disorder, orgasm disorder, sexual pain, and vaginal spasm. In recent years, an increasing number of studies have reported that women with endometriosis have a lower sexual quality of life, such as sexual functioning and satisfaction [20,21].

Endometriosis can have a significant effect on various aspects of women’s lives, including their social and sexual relationships, work, and study (De Graaff et al., 2013) [22,23]. In addition to the classic symptoms, women with endometriosis are more likely to develop depression and anxiety (Lorençatto et al., 2006; Pope et al., 2015) and their quality of life may be affected by pain, the emotional impact of sub-fertility, a possible recurrence of the disease and uncertainty about the future related to repeated surgeries and the long duration of medical therapy (Berek et al., 2012).

Few studies in women have evaluated the negative effect of the endometriosis disease on sexual function because of the pain (Ferrero et al., 2005; Fritzer et al., 2013; De Graaff et al., 2016, 47, 48); another analysed the effect in couple’s relationship [1]. 

Despite dyspareunia, as well know, plays an important role as an etiologic factor of sexual dysfunction [24,25], which is source of low self-esteem and negative effects on interpersonal relationships [22], there are other factors not necessarily related to severity of the disease or level of tissue invasion (that include women’s age, mental health, or social or cultural correlates) have been also reported [26,27,28,29,30]. Women with endometriosis report more depression and anxiety symptoms [31], which might in turn impact sexual function. 

Therefore, we aimed to assess the sexual function of Spanish women with endometriosis in relation with sociodemographic (unemployment and age), clinical (deep endometriosis) and psychological factors (depression) that influence sexual function (sexual arousal, lubrication, and orgasm) and sexual satisfaction (sexual pain).

## 2. Materials and Methods

Between 1 January 2021 and 30 June 2021, a multicentre cross-sectional study was performed to assess sexual function in a sample of Spanish women diagnosed with endometriosis, using the validated Female Sexual Function Inventory (FSFI) [30,31]. The diagnosis of endometriosis was made in accordance with the guidelines of the European Society of Human Reproduction and Embryology (ESHRE), based on the visual detection of endometriosic lesions during previous surgeries, anatomopathological tests, and typical ultrasonographic features of endometriosis [32]. Patients with suspected endometriosis, without surgical or ultrasound confirmation, were excluded from further analysis. The study was performed simultaneously in three reference hospitals in Spain (University Hospital of Jerez, University Hospital of Murcia, and University Hospital of Málaga). In the study period a total of 368 women with endometriosis attended the reference units for control. By consecutive sampling, a representative group of 196 were recruited (5% standard error and 95% confidence level, with a power of 0.95). During an on-site medical visit, women were invited to complete an anonymous questionnaire, containing 92 questions, which took approximately 20 min to complete. The questionnaire included an informed consent, the Spanish validated version of the FSFI, the validated Spanish-language version of Beck’s depression scale (BDS), the State-Trait Anxiety Inventory (STAI), the Short Form Health Survey (SF-12), and the 10-item short form of the Connor–Davidson Resilience Scale (CD-RISC-10). We also included a series of questions about socioeconomic status, physical and mental health status, and obstetric background information. A blinded review of medical records was conducted for every participant was performed by the authors of this research, so that all data regarding the evolution of endometriosis was collected a second time, before analysing the answers to scale. All participants were asked to sign an inform consent sheet. Inclusion criteria: 18 years or older, sufficient reading skills to complete self-report instruments, and symptomatic endometriosis at the time of assessment. Acceptance of the data protection laws and the consent form to participate in the study were included.

### 2.1. Instruments

The FSFI was developed to analyse domains of sexual function during the previous four weeks; it is still one of the most widely used scales to evaluate sexual dysfunction in women. The questionnaire evaluates 19 items on a Likert-type scale, with each item evaluated from 0 to 5, according to the level of agreement or disagreement. Six domains were considered, i.e., sexual desire, arousal, lubrication, orgasm, satisfaction, and pain. For domain scores, individual items were added, multiplying the sum by a specific correction factor. FSFI scores range between 2 and 36, with greater values indicating better sexual function. The cutoff score for normal sexual function is 26.5 [33,34].

The Beck´s Depression Inventory (BDI) is a self-administered questionnaire consisting of 21 Likert-type questions. Cut-off points were designed to enable the classification of respondents into four groups: 0–13: minimum depression; 14–19: mild depression; 20–28: moderate depression; 29–40: severe depression; and more than 40: extreme depression. This was also validated in Spanish [35]. 

The State-Trait Anxiety Inventory [36] is also a self-administered questionnaire, and validated in Spanish as well [37], composed of two scales and scores that define different levels of state or trait anxiety, i.e., low (between 0 and 30), moderate (between 30 and 44), and high (over 45). 

The SF-12 Health Questionnaire [38,39] is a generic scale that provides a profile of one’s health and is applicable to both patients and the general population. It is comprised of 12 questions (items) that assess both positive and negative states of health. The SF-12 is one of the most widely used instruments for assessing self-reported health quality of life (HRQOL). It covers eight health domains, including physical and mental health, and scores ranging from 0 to 100, with higher scores indicating better quality of life and health. It has been validated in several languages, including Spanish [40]. CD-RISC10 [41], and is an improved self-administered questionnaire of 10 items from the original 25-item-scale of Connor and Davidson [42]. The score ranges from 0 to 40 in the short form, and has been translated and validated into Spanish [43].

The sociodemographic questionnaire included variables such as city of residence, women´s ages, academic level, employment status, and family incomes—as well as clinical variables regarding obstetric and medical background, family planning, endometriosis diagnosis date, type of treatment, number of surgeries, and stage of the disease, which were also recorded.

### 2.2. Population

The survey was completed by 196 women with endometriosis who were attended in three Spanish reference hospitals. No difference was found in the number of cases contributed by each referral centre. Mean age was 39.5 years (SD 6.8), with an average of 7.1 years after diagnosis of endometriosis. Moreover, 103 women (52.5%) were nulliparous, and 16.8% reported difficulties getting pregnant at the time of recruitment, with 21.4% having used assisted reproductive techniques. Most participants were under medical treatment (78.1%), and 51.8% reported having undergone laparoscopic (35.9) or laparotomic (15.9%) surgery. Most of the women (57.6%) had been diagnosed with deep endometriosis, 38.9% with limited ovarian endometriosis, and 21.9% suffered with adenomyosis. We observed some comorbidities among participants: depressive mood (40.4%), asthma (12.7%), and hypothyroidism (8.6%) were the most prevalent. The most reported symptom was pain. Dysmenorrhea (83.4%), abdominal pain (73.6%), dyschezia (47.2%), dyspareunia (32.6%), and dysuria (29.2%) were the principal clinical manifestations of pain, and according to the visual-analogue scale (VAS) the pain level ranged from moderate to very severe, with a mean of 7.5/10 (SD 2.3) for dysmenorrhea, 6.4/10 (SD 2.2) for abdominal pain, 5.5/10 (SD 2.5) for dyschezia, 5.5/10 (SD 2.6) for dyspareunia, and 4.9/10 (SD 2.5) for dysuria. Table 1 shows the sociodemographic features of the sample.

### 2.3. Statistical Analysis

We performed a first analysis of the frequency distribution of independent variables. For bivariate analyses, we used the independent sample *t*-test to compare mean values in the two groups of women; when the number of groups was greater than two, we used single-factor ANOVA. The conditions of homoscedasticity were evaluated using Levene’s test. The size effect was measured using Cohen’s D statistic. To analyse the relationship of the scores between the FSFI and other psychometric tools, or other quantitative variables (normally distributed), e.g., age or years of evolution of the disease, we used Pearson´s correlation coefficient, calculating the level of significance.

We studied the consistency of FSFI scores with Cronbach´s alpha coefficient and performed an exploratory factor analysis (EFA) on factor structure, while validating the scale in our population. The EFAs were conducted by analysis of the principal components of the scale, with the Varimax rotation method to identify latent factors of observed variance.

To predict low FSFI scores, we used logistic regression models based on independent sociodemographic, health-related, and emotional variables. To predict the influence of psychometric scales (BDI, STAI, SF-12, and CD-RISC10) on FSFI results, we used multiple linear regression. The collinearity between factors was analysed to avoid inclusion of correlated variables in the model. The models were constructed using a stepwise regression procedure, including the psychometric scales and the clinical and sociodemographic variables that were shown to be significantly associated. Additionally, structural equation modelling (SEM) analyses with correlated factors were performed using the maximum likelihood estimator. Four fit indices were selected a priori to assess them: Comparative Fit Inventory (CFI), Tucker–Lewis index (TLI), Standardized Root Mean Square (SRMS), and Root Mean Square Error of Approximation (RMSEA). Acceptable model fit was defined by a CFI ≥ 0.90, Tucker–Lewis index ≥ 0.90, SRMR or RMSEA values ≤ 0.08 [44,45]. Based on these criteria, the best-fitting final model was selected. Statistical analyses were performed using SPSS v. 25 software (Chicago, IL, USA); p values of 0.05 were considered significant.

## 3. Results

### 3.1. Validation of FSFI in the Sample

We found a Cronbach’s alpha score of 0.86. Kaiser–Meyer–Oltkin and Barlett´s test revealed sampling adequacy to perform an exploratory factor analysis (EFA) (KMO 0.86; Chi-Square 3131.5, 171 df; *p* < 0.0001). We found that by using eigenvalues equal to or greater than 1.0 as criteria for factor extraction, four factors were identified explaining 78.9% of the total variance. These four factors were similar to those reported in the original FSFI Spanish validation [40,46], i.e., desire/arousal, lubrication, orgasm, satisfaction, and pain. We performed a second EFA, restricting factor extraction in a six-factor structure, based on the original authors´ description [29]. This six-factor model explained the 86.5% total variance and the minimum eigenvalue of 0.67.

### 3.2. FSFI Scores

The mean FSFI score for the sample was 22.5 (SD 6.6), with 20.9 and 26.9 being the 25th and 75th percentiles, respectively. We found that 72.1% of participants scored under the accepted cut-off point and suffered from impaired sexual function. The distribution of FSFI scores and its six dimensions are shown in Table 2.

We found differences in the FSFI global score and its dimensions, according to sociodemographic features, so that women in secondary and university studies scored significantly lower (FSFI: 23.5, SD: 4.5) than women with primary or less studies (FSFI: 27.15 (SD: 1.75); F: 4.13; *p* = 0.007). Married women scored higher (FSFI: 24.4 (SD:4.3)) than singles (FSFI: 20.75 (SD: 4.9); Cohen’s D = 0.29; F: 6.67; *p* = 0.002). Regarding employment status, we found that self-employed women scored lower (FSFI 20.2 (SD: 6.6)) than salaried women (23.8 (SD: 4.0)) or those supported by state unemployment benefits (FSFI: 28.5 (SD: 2.5); F: 0.007; *p* < 0.0001). On the other hand, women with children were found to score significantly higher (FSFI: 23.4 (SD: 5.7)) than women without offspring (FSFI: 21.3 (SD: 7.5); Cohens D = 0.52). According to the type of disease, women with deep endometriosis (FSFI: 21.64 (SD: 4.2)) and adenomyosis (FSFI: 23.2 (SD: 4.7)) scored significantly lower than those with ovarian endometriosis (FSFI: 25.0 (SD: 4.5); Cohen’s D = 0.51; F: 4.9; *p* < 0.001). Other clinical features influenced FSFI results, so that women with dyspareunia (FSFI: 14.0, (SD: 8.3); Cohen’s D 0.54) or dysuria (FSFI: 20.37 (SD: 7.4); Cohen’s D = 0.21) as main symptoms had significantly poorer sexual function compared to participants with other kind of symptoms, like dysmenorrhea or dyschezia. We found that those women with dyspareunia scored significantly lower in pain (r = −0.44, *p* < 0.001) and orgasm (r = −0.20, *p* < 0.003) FSFI domains, but presented a positive significant correlation with the sexual desire domain (r = 0.25, *p* = 0.002). We observed a negative significant correlation between FSFI scores and the number of laparotomies that women underwent (r = −0.67, *p* < 0.001).

### 3.3. Mental Health, Mood Disorders, Resilience and Sexual Function

Reliability measures for the scales can be found in Table 3. The mean score in the mental health domain of SF-12 was 47.44, with 40.5, 49.5, and 55.8 being first, second, and third quartiles. We observed a significant correlation between the scores of SF-12 mental health and FSFI scores (r = 0.163, *p* = 0.036). The SF-12 mental health scores were significantly lower (SF-12 mean score = 45.0) in women with FSFI scores under the cut-off value for sexual dysfunction (t = 2.9, Mean difference = 4.0; Cohen’s D 0 0.4; *p* = 0.004). Mean BDI score was 3.8 (SD: 3.2). We found that 12.6% of participants scored for mild depression. In general, anxiety levels were also low with mean values of 22.7 (SD: 11.2) and 23.5 (SD: 11.4) for state and trait anxiety, respectively. Among all participants, 24.8% and 24.2% scored at least moderate state or trait anxiety. We observed that mood disturbances influenced sexual functioning. We found a significant negative correlation between BDI scores and FSFI (r = −0.24; *p* < 0.001). Women scoring over the cut-off point for mild depression were found to score more frequently under the cut-off point for a poor sexual function, so that 92.6% of depressed women versus 75% of non-depressed participants scored on the FSFI under 26.5 (Chi-square: 25.8; *p* < 0.0001). Additionally, women who scored for mo-derate state or trait anxiety were found to have a poorer sexual function. We observed that 60.8% of women with moderate or severe state anxiety versus 35% of women without it scored under the FSFI cut-off (Chi-square: 13.6, *p* < 0.001); likewise, 59.2% of women with moderate or severe trait anxiety scored for poor sexual function, while only 35.7% of women with lower scores did (Chi-square: 11.9; *p* < 0.0001). The associations between FSFI domains and anxiety levels are shown in Table 4 and Table 5.

Regarding resilience on CD-RIS-10 scores, we found significant correlations with BDI scores (r = −0.4; *p* < 0.001), trait anxiety scores (r = −0.28; *p* < 0.001), physical health (r = 0.18; *p* < 0.001), and mental health (r = 0.34; *p* < 0.001). We found that resilience (CD-RISC-10) scores correlated negatively with FSFI (r = −0.185; *p* < 0.01). The mean CD-RISC-10 score in the sample was 29.2 (SD: 7.1). We observed that participants with very low levels of resilience (scoring under the first quartile) got significantly higher FSFI scores than those women scoring higher on CD-RISC-10 (FSFI mean score was 26.5 (SD: 1.3) in those with very low resilience, and 23.9 (SD: 4.5) in the group of women with higher CD-RISC-10 scores (Cohen’s D = 0.21; F = 3.85; *p* < 0.05).

### 3.4. Multivariate Analysis

**Figure 1:**
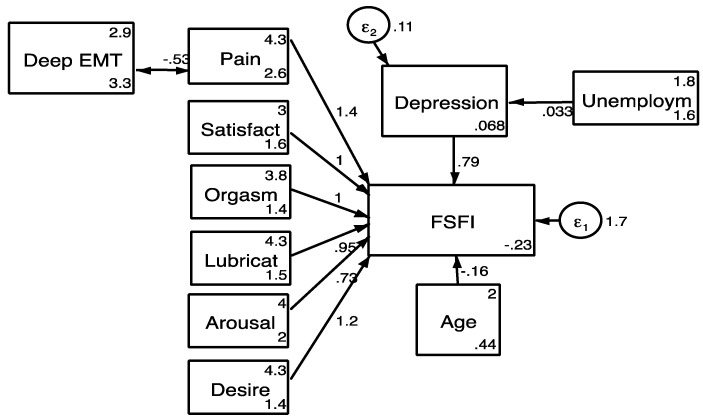
Best fitting model. SEM. Chi2 9.62 model vs. saturated, *p* = 0.474; Root mean squared error of approximation 0.00; Akaike’s information criterion, 5686.080; Bayesian information criterion, 5911.913; Comparative fit index, 1.000; Tucker–Lewis index, 1.002; Coefficient of determination 0.937.

Logistic regression showed that age, employment status, and deep endometriosis were predictors of sexual function, with significant odds ratios for sexual dysfunction (Table 6). The stepwise multiple linear regression analysis showed that the predictive model for FSFI score included BDI scores as single predictive variable (Table 7). The final best fitting model after the SEM analysis, as well as all goodness of fit parameters, can be seen in Figure 1.

## 4. Discussion

Endometriosis is a common gynaecological disease, which occurs in women of childbearing age. Sexual function and quality of sexual life of patients are affected by varying degrees in disease and treatment as show aur results. Our study used the Spanish version of the FSFI to determine sexual function of patients with endometriosis.

Sexual dysfunction refers to the fact that woman cannot participate in their desired sexual life because of reasons, such as unmet sexual desire, arousal disorder, orgasm disorder, and sexual pain. Endometriosis can cause more serious sexual pain, which is because of sexual activity. This results in an increase in tension of the uterine sacral ligament, displacement, deep sexual pain, and pain after sexual intercourse. Montanari et al. [45] found that patients with deep infiltrating endometriosis had impaired sexual function and sexual pain; vaginal ectopic lesions were related to sexual dysfunction.

Long-term illness can cause anxiety, depression, and other psychological symptoms. Women with sexual dysfunction show inferiority, a lack of self-confidence, and fear of pain caused by sexual intercourse, which affect the feelings of couples and seriously affect women’s health and quality of life.

Despite endometriosis being a benign gynaecological disease, it can affect female sexual function to a certain extent. This suggests that clinicians and nurses need to take active measures to improve the quality of sexual life of patients with endometriosis [47].

Therefore, in clinical practice, patients with endometriosis should be offered targeted psychological counselling, and their coping styles should be enhanced. In particular, these patients should be informed that they can return to a normal sexual life after 3 months of follow-up evaluation. Additionally, patients should understand the anatomical structure and physiological function of the female reproductive system, surgical methods, and the effect of treatment. This could reduce the unnecessary psychological burden and increase self-efficacy [48].

This article presents results of sexual function in a sample of 196 women with endometriosis from three specialized reference units for its treatment in Spain and shows clinical and psychosocial correlates. We found an overall poor sexual function in most participants, with 72.1% scoring on FSFI for sexual dysfunction. Overall, sexual satisfaction, arousal, pain, and orgasm were the most severely deteriorated FSFI domains. Although physical sexual pain is one important factor that influences sexual function of women with endometriosis, our results show that impairment is multifactorial. Factors such as not having a job increased the occurrence of dyspareunia, dyschezia, and dysuria, with those using analgesics having significantly higher scores.

This issue was addressed in a recent metanalysis in which higher rates of dyspareunia in women with endometriosis did not predict sexual distress, and metacognitive beliefs were more influential on sexual distress than pain [47]. Exploratory hierarchical regression analyses revealed that for women, age and relationship satisfaction (both treated as covariates), as well as depression, emerged as statistically significant correlates of sexual function (i.e., women who were older and reported greater levels of depression and less satisfaction with their current relationship indicated poorer sexual functioning). These factors are in no way exhaustive, but they provide important insight into the sociocultural influence of female sexual desire. While not easy, lifestyle habits and relational components can be adjusted.

Our predictive model shows that deep endometriosis significantly affects sexual function more than ovarian endometriosis or adenomyosis, producing pelvic pain probably due to compression or infiltration of the endometriotic implants in the sub-peritoneal space [22,49]. According to these results, reduction of sexual pain should be the first treatment for women with sexual dysfunction associated with deep endometriosis, using a combination of medical and surgical therapies.

However, the effect of psychosocial factors needs to also be addressed. It is widely acknowledged that endometriosis can result in considerable psychological and social difficulties, for example depression, anxiety, and difficulties in carrying out normal daily activities [22,23]. Our results show that depression plays an important role in the sexual functioning of these women, and that psychological support should be provided in endometriosis units, although some women may feel their psychological needs are being met or may feel they do not need this support [22]. Published literature shows that psychological counselling and support should be focused on helping women integrate endometriosis into their history, not only in the management of pain [50,51,52]. In our predictive model, resilience did not show as a predictive factor of sexual function. Resilience protected women from being depressed, but it did not seem to have a direct effect on sexual function scores. As previously reported, endometriosis impacts working lives and household incomes [1] with potential effects on mood and mental health. In our study, employment status predicted depression levels as well as sexual function. Women with endometriosis have significantly lower quality of life (QoL) than the general female population with younger women having more symptoms and lower QoL [53]. In our study, ages ranged from 24 to 51 years, which could also negatively affect sexual function and arousal.

Our study is multicentric research, which contributes a multidimensional perspective of sexual function in women with endometriosis, however, some limitations must be considered. The sample is relatively small with low power and lacks a control group as it is a cross-sectional study; therefore, it was not possible to report causal relationships between sexual function and all other factors. In addition, we did not study sexual function in women´s partners.

## 5. Conclusions

Women with endometriosis report a significant effect of the disease on sexual function. We found impaired sexual function in the population of Spanish women with endometriosis. The best fit predictive model included deep endometriosis, depression level, age, and unemployment, which were strong predictive factors.

## Figures and Tables

**Table 1:** Sociodemographic and clinical features of the sample.

Type of EMT	*n* (%)	
Ovarian	77 (38.9)	
Deep	23 (11.6)	
Adenomyosis	6 (3.0)	
Deep and ovarian	53 (26.8)	
Deep and adenomyosis	17 (8.6)	
Deep, ovarian, and adenomyosis	20 (10.1)	
**Diagnosis**		
Clinical	121 (55.0)	
Laparoscopic	73 (33.1)	
Laparotomic	26 (11.8)	
**Current treatment**		
Yes	172 (78.1)	
No	48 (21.8)	
**Academic level**	***n*** **(%)**	
None	6 (3.0)	
Primary	25 (12.4)	
Secondary	69 (34.2)	
University	102 (50.5)	
**Incomes**		
EUR <600	33 (17.1)	
EUR 600–1200	82 (42.5)	
EUR 1200–3600	72 (37.3)	
EUR >3600	6 (3.1)	
**Source of incomes**		
Salaried	130 (69.5)	
Self-employed	19 (10.2)	
Help from relatives	8 (4.3)	
Subsidies	30 (16)	
**Working status**		
Active	140 (70.0)	
Housewives	10 (5.0)	
Unemployed	32(16.0)	
Sick leaves	14 (7.0)	
Retired	2 (1.0)	

**Table 2:** Score distribution.

		Total Score	Sexual Desire	Sexual Arousal	Vaginal Lubrication	Orgasm	Sexual Satisfaction	Sexual Pain
Mean		22.5	4.1	3.8	4.1	3.6	2.7	3.2
Std. Deviation		6.7	1.4	1.5	1.5	1.4	1.4	1.8
Percentiles	25	20.9	3.6	3.0	4.2	3.6	1.6	3.0
	50	23.8	4.2	3.9	4.5	3.6	2.8	4.4
	75	26.9	4.8	4.8	5.1	4.4	3.6	4.8

**Table 3:** Reliability measures for the scales.

Scales	Crombach’s Alpha (Inter-Item Covariance)	Scales	Crombach’s Alpha (Inter-Item Covariance)
FSFI	0.86 (Cov 0.57)	DBI	0.78 (Cov 0.02)
STAI state	0.92 (Cov0.28)	SF12	0.86 (Cov 0.24)
STAI trait	0.91(Cov 0.27	CD-RISC-10	0.91 (Cov 0.46)

**Table 4:** Mean scores and standard deviation of FSFI domains according to anxiety levels (low versus moderate to severe). NS, not significant.

	State Anxiety			Trait Anxiety		
	Low *N* = 118	High *N* = 80		Low *N* = 112	High *N* = 86	
Sexual desire	4.25 (1.09)	4.28 (1.28)	F = 0.029 *p* < 0.865 D = 0.25	4.12 (1.15)	4.46 (1.17)	F = 3.98 *p* < 0.047 D = 0.29
Sexual arousal	3.75 (1.33)	4.30 (1.48)	F = 6.75 *p* < 0.010 D = 0.39	3.75 (1.28)	4.24 (1.52)	F= 5.85 *p* < 0.016 D = 0.35
Lubrication	4.33 (1.26)	4.21 (1.22)	F = 0.410 *p* < 0.523 D = 0.01	4.28 (1.21)	4.29 (1.28)	F = 0.004 *p* < 0.95 D = 0.0
Orgasm	3.69 (1.07)	3.88 (1.40)	F = 0.975 *p* < 0.325 D = 0.01	3.70 (1.09)	3.85 (1.35)	F= 0.678 *p* < 0.411 D = 0.12
Sexual satisfaction	2.72 (1.24)	3.28 (1.26)	F= 8.17 *p* < 0.005 D = 0.44	2.72 (1.23)	3.21 (1.29)	F = 6.75 *p* < 0.01 D = 0.39
Sexual pain	4.61 (1.58)	3.91 (1.61)	F = 8.76 *p* < 0.003 D = 0.44	4.59 (1.58)	4.02 (1.62)	F = 5.78 *p* < 0.047 D = 0.35

**Table 5:** Correlation indexes for FSFI domains and other scales. Pearson’s correlation coefficients. Significance level *p* < 0.05. NS, not significant.

Dimensions	BDI	STAI Trait	STAI State	SF-12 Mental	SF-12 Physsical	CD-RISC
Sexual desire	0.960 *p* < 0.194	0.211 *p* < 0.04	0.018 *p* < 0.806	−0.241 *p* < 0.001	−0.085 *p* < 0.241	−0.114 *p* < 0.119
Sexual arousal	0.288 *p* < 0.001	0.315 *p* < 0.001	0.067 *p* < 0.363	−0.311 *p* < 0.001	−0.069 *p* < 0.348	−0.229 *p* < 0.002
Lubrication	0.193 *p* < 0.001	0.90 *p* < 0.226	0.102 *p* < 0.169	−0.019 *p* < 0.795	−0.081 *p* < 0.275	−0.014 *p* < 0.849
Orgasm	0.243 *p* < 0.001	0.167 *p* < 0.025	0.085 *p* < 0.259	−0.065 *p* < 0.385	0.01 *p* < 0.898	−0.070 *p* < 0.353
Sexual satisfaction	0.243 *p* < 0.001	0.280 *p* < 0.001	0.018 *p* < 0.808	−0.472 *p* < 0.001	−0.070 *p* < 0.358	−0.304 *p* < 0.001
Sexual pain	−0.093 *p* < 0.217	−0.181 *p* < 0.014	0.091 *p* < 0.219	0.278 *p* < 0.001	0.189 *p* < 0.01	0.196 *p* < 0.008

**Table 6:** Logistic regression model.

	B	S.E.	Wald	df	Sig.	Odds Ratio	OR 95% C.I.	OR 95% C.I.
							Lower	Upper
Age < 35 years	1.49	0.82	3.30	1	0.01	4.45	0.89	22.27
Age 35–45 years	1.10	0.62	3.11	1	0.077	3.00	0.88	10.20
Unemployment	1.54	0.68	5.13	1	0.02	4.70	1.23	17.97
Deep EMT	0.97	0.43	5.00	1	0.002	2.65	1.12	6.25
Constant	2.26	1.30	3.03	1	0.08	0.104	-	-

**Table 7:** Linear multiple regression. Best fitting model R square 0.694. VIF, variance inflation factor.

Model	Unstandardized Coefficients		t	Sig.	95% Confidence Interval for B		Collinearity Statistics	
	B	Std. Error			Lower Bound	Upper Bound	Tolerance	VIF
(Constant)	21.8	0.818	26.7	0.00	20.12	23.612	1	1
BDI score	1.5	0.258	5.8	0.00	0.95	2.052	-	-
